# Determinants of Ownership and Utilization of Insecticide-Treated Bed Nets for Malaria Control in Eastern Ethiopia

**DOI:** 10.1155/2012/235015

**Published:** 2012-11-07

**Authors:** Sibhatu Biadgilign, Ayalu Reda, Haji Kedir

**Affiliations:** ^1^Department of Epidemiology and Biostatistics, College of Public Health and Medical Sciences, Jimma University, P.O. Box 24414, Jimma, Ethiopia; ^2^Department of Public Health, College of Health Sciences, Haramaya University, P.O. Box 1014, Harar, Ethiopia; ^3^Addis Continental Institute of Public Health, P.O. Box 1014, Addis Ababa, Ethiopia

## Abstract

*Background*. Malaria remains a major cause of mortality and morbidity in the world, and particularly in sub-Saharan Africa. *Objectives*. The aim of this study was to determine ownership and utilization of ITNs among households with children under five in the previous night. *Methods*. A community based cross-sectional study was conducted in Gursum district in Eastern Ethiopia. A total of 335 households were surveyed using a pretested structured questionnaire administered though house-to-house interviews. *Results*. Household ownership for at least one mosquito net and use of nets were 62.4% (95% CI 57.2–67.6%) and 21.5% (95% CI 17.1–25.9%), respectively. Households who received or were told about ITN in the last 6 months were three times more likely to have used it than those who were not (OR 3.25; 95% CI 1.5–7.10). Households whose heads were engaged as a farmer (adjusted OR 0.137; 95% CI: 0.04–0.50) and housewife (OR 0.26; 95% CI: 0.08–0.82) were less likely to use ITN than those of other occupations. *Conclusion*. The findings indicate low ITN ownership and utilization among the households. Intensive health education and community mobilization effort should be employed to increase the possession and proper utilization of insecticide treated bed nets.

## 1. Background

The World Health Organization (WHO) estimated that the number of cases of malaria rose from 233 million in 2000 to 244 million in 2005 but decreased to 225 million in 2009. The number of deaths due to malaria is estimated to have decreased from 985,000 in 2000 to 781,000 in 2009 [1]. Malaria remains a major public health problem particularly in sub-Saharan Africa [2] which accounts for about 90 per cent of all malaria deaths, most among children under age five [3]. Malaria-related mortality, morbidity, and economic loss could be averted if the available effective preventive and treatment interventions were made universally accessible to those in need [4]. In Africa about one out of every twenty children is likely to die of malaria-related illness before their fifth birthday [5].

In Ethiopia, malaria transmission is highly seasonal and often takes epidemic forms where significantly higher malaria morbidity and mortality occurs during the peak transmission season from September to December. Even though all age groups are at risk of developing severe malaria, children and pregnant women are the most vulnerable [6]. Approximately 75% of Ethiopia's landmass is malaria endemic [7–9]. The coverage and proper utilization of the most promising malaria preventive measure, insecticide-treated bed nets (ITNs), in the country is also limited due to lack of sustainable distribution and issues related to replacement of nets, seasonality of malaria, and poor knowledge of the community with regard to the link between mosquitoes and malaria [10].

The Roll Back Malaria (RBM) has identified under-five children as one of the highest risk groups for malaria, and one of the strategies set to fight malaria in this group is to increase utilization of mosquito nets [11]. The use of insecticide-treated mosquito nets (ITNs) is a cost-effective intervention to reduce child mortality and maternal anemia where malaria imposes an important disease burden [12]. Roll Back Malaria (RBM) campaign targets to have 80% of pregnant women and children under five covered by insecticide-treated bed nets (ITN) by 2010 [13]. 

Identification of awareness gaps, monitoring of behavioral changes on malaria disease recognition and use of preventive and control measures such as the use of ITNs are a priority area for the government of Ethiopia with a special emphasis on increasing coverage and use of ITNs under national malaria guidelines [14]. Few studies assessed the ownership and use of ITNs by households of under-five children. This paper describes household ownership and utilization of ITNs in the previous night among this vulnerable group in a malaria-prone rural district in eastern Ethiopia.

## 2. Methods

### 2.1. Study Setting and Participants

A community-based cross-sectional study was conducted in Gursum district, eastern Ethiopia in January 2008. This rural town district had a total population of 12,027. It was purposely selected for this study because it is among the major malaria-prone areas of eastern Ethiopia. The mean/median altitude of the study district ranges from 1200 to 2950 meters above sea level. The source population included all households with children under five years of age. Households were randomly selected from a list provided by the district administration. The sample size was calculated by using the formula for a single population proportion considering the assumption 95% CI and 5% margin error and prevalence of ITN use as 50%. This gives a final sample size of 384. Health extension workers (HEW) distributed the Long lasting insecticide-treated net (LLIN) to the community at their vicinity/households in a procedure of free mass distribution campaigns. The study was conducted from February to March 2006. 

### 2.2. Questionnaire and Data Collection

Data were collected using a pretested structured questionnaire prepared in English and then translated into the local language of Oromiffa. The questionnaire was adopted from instruments developed by the Roll Back Malaria (RBM) partnership monitoring and evaluation reference Group by the WHO and UNICEF [15]. It included variables related to sociodemographic characteristics, number of household members, net possession, net utilization, and so forth. Pretest was carried out on 5% of the households. Necessary modifications were made thereafter. Final year public health professionals administered the questionnaire through house to house visits. Information was primarily collected from the heads of the households (father or mother) or from an adult household member in case this was not possible. 

### 2.3. Operational Definitions

Households were considered ITN owners if they had at least one ITN at the time of the interview. Children that were reported to have slept under an ITN in the night prior to the survey interview were considered as users.

### 2.4. Statistical Analysis

Data were checked for completeness and consistency. The data were entered at once by a trained data manager. Coded data were entered, cleaned, and analyzed using SPSS version 16.0 for Windows (SPSS, Chicago, IL, USA). Descriptive summaries (frequencies and proportions) and univariate analysis were calculated. Multivariable logistic regression analysis was used for determining ITN ownership and utilization as the main outcome variables. Unadjusted and adjusted odds ratios (AOR) and their corresponding 95% confidence intervals (CI) were used to examine the strength of association. *P* values of less or equal to 0.05 were considered significant.

### 2.5. Ethical Clearance

Ethical clearance was obtained from Haramaya University, Faculty of Health Sciences Institutional Research Ethics Review Committee. Verbal consent was obtained from individual respondents.

## 3. Results 

### 3.1. Sociodemographic Characteristics of the Study Population

A total of 335 households participated in this study with a response rate of 88%. The mean (SD) family size of the households was 5.45 (2.85) with a median of 5. The majority (247, 73.7%) of the respondents were females, while 228 (68.1%) were illiterate (see Table 1). 

### 3.2. Insecticide-Treated Bed Net (ITN) Ownership and Utilization

Household ownership for at least one mosquito net in the surveyed households was found to be 209 (62.4%; 95% CI 57.2–67.6%). Eighty seven (41.6%) and 116 (55.5%) households owned one and two nets, respectively. The nets were obtained from the district health office (96, 45.9%) and the local health center (32, 15.3%) for free and from other sources (52, 24.9%). Sixty households (28.7%) and 90 (43.1%) reported to have obtained the ITNs 6–12 months and 13–24 months, respectively, prior to the time of the survey (Table 2). Forty five (21.5%; 95% CI 17.1–25.9%) of the surveyed households utilized bed nets.

### 3.3. Knowledge and History of Malaria in the Past One Year

Among the households studied, in 81 (24.2%) at least one family member had symptoms of malaria in the past one year. The signs and symptoms reported were fever 80 (23.9%), chills (77, 23.0%), and headache (75, 22.4%). The majority got treatment from government health facilities (65, 81.2%) and private clinics (12, 15.0%). The most important sources of information on malaria cited by respondents were mass media (148, 48.5%), health workers (87, 28.5%), local village leaders (16, 5.2%), health extensions workers (24, 7.97%), and neighbors (30, 9.83%). About 58 (17.3%) households had a family member that traveled outside the district in the month prior to the survey (Table 3).

### 3.4. Determinants of ITN Ownership and Utilization

Households whose heads were farmers were five times more likely to have owned ITNs than those whose heads were traders (AOR 5.11; 95% CI 1.88–13.94). Households with respondents that knew the cause of malaria were twice more likely to have owned ITNs than those who did not know (AOR 2.64; 95% CI 1.31–5.30). Those who ever heard about ITN were seven times more likely to have owned one than those that did not (AOR 7.56; 95% CI: 1.95–29.28). Households whose family size was less than 3 were 77% less likely to have owned ITNs than those of seven or more family size (AOR 0.23; 95% CI: 0.10–0.52) (Table 4). 

Those households who received or were told about ITN in the last 6 months prior to the survey were three times more likely to have used it than those who were not (AOR 3.25; 95% CI 1.5–7.10). Households whose family size was less than or equal to four were two times more likely to have used ITNs than those whose family size of greater than four (AOR 2.32; 95% CI 1.06–5.05). Occupationally, those households whose heads were engaged as a farmer (AOR 0.14; 95% CI: 0.04–0.50) and housewife (AOR 0.26; 95% CI: 0.08–0.82) were less likely to use ITNs than those of traders (Table 5). 

## 4. Discussion 

The World Health Assembly and the Roll Back Malaria (RBM) partnership in 2005 set the goal of reducing the numbers of malaria cases and deaths recorded in 2000 by 75% or more by the end of 2015 [1]. There has been a noticeable increase in international funding for malaria control in the past decade. This increased financing has led to tremendous progress in increasing access to insecticide-treated mosquito nets (ITNs). It is reported that by the end of 2010, approximately 289 million ITNs were delivered to sub-Saharan Africa, about enough to cover 76% of the 765 million persons that are at risk of malaria [1].

This study aimed to assess ownership and utilization of bed nets in a rural town of eastern Ethiopia. Our finding indicates an ITN ownership of 62.4% (95% CI 57.2–67.6%). It is estimated that 42% of households in Africa owned at least one ITN in mid-2010, and that 35% of children slept under it [1]. The percentage of children using ITNs is still below the World Health Assembly target of 80% partly because up to the end of 2009, ITN ownership remained low in some of the largest African countries. 

Low rates of use reported in surveys are primarily due to lack of sufficient nets to cover all household members; household survey results suggest that most (80%) of the available ITNs are used [1]. According to a malaria indicator survey conducted in 2007 among 5,083, in Ethiopia, 65.6% of households residing in malaria-prone areas owned at least one ITN [16]. Similarly, in malaria-prone areas, ITN use by children under five years and pregnant women has remarkably leapt from 2.8% and 1.6% to 41.2% and 42.5%, respectively, in 2005 [17]. In MIS 2007, a total of 14,663 participants in 3,353 households owned nets. Despite an increase in the proportion of households owning at least one net, a lower proportion of participants (50.9%) reported using nets the previous night during MIS 2007 [18]. Household possession for at least one mosquito net in the surveyed households was found to be 748 (90.3%) and household possession for at least one insecticide-treated bed net was found to be 708 (85.5%) [19]. In our study, 62.4% of households had at least one ITN. In a study carried out by Baume et al., of 857 surveyed households, 780 (91%) owned at least one ITN in Oromia and Amhara regions of Ethiopia. 63% of ITN-owning households owned more than one ITN [20]. Household ownership of any net was 23.9% (95% CI 22.8%–25.1%) and 10.1% for ITNs (95% CI 9.2%–10.9%). Utilization of any net by children under five was 11.5% (95% CI 10.4%–12.6%) and 1.7% (95% CI 1.3%–2.2%) for ITN [21]. The utilization of ITN in our study is 45 (21.5%). Fegan et al., found that consistent use of ITNs can reduce malaria transmission by up to 90% and avert as much as 44% of all-cause mortality in their study of under-five children in rural Kenya [22]. 

Occupational status of the household head and family size were among the factors affecting the ownership of ITN in our study. Similar findings have been documented in other studies. In one study in Ethiopia, government employees and self-employed traders were less likely to own a net [19]. In our study, family size was associated with the possession of net. Similarly in Tanzania a unit increase in family size increased the odds of ownership of a net more than twice while controlling for all other variables, where for households who had at least one under-five child the odds of owning any net was about 60% higher than those with no under-five children [21]. In another study, women's and head of household's education, head of household's occupation, marital status, household size, household wealth, living in rural areas, and expenditure on other malaria prevention products and practices were found to be associated with ITN ownership [23–25].

A strong association remained between using ITNs, owning a radio, and living close to a health institution [26]. In addition, households' desire for mosquito avoidance and correct knowledge of malaria transmission were reported to be strong determinants of ITN usage [27, 28]. The acquisition and usage of untreated mosquito nets was low and nil for ITNs and very few people had heard about ITNs [29]. The results are also similar to the findings in Mozambique, where only 3% of people had heard about ITNs and 9% used treated or ordinary nets [30]. In one study in Ethiopia, it was found that the role of health workers was minimal in delivering health education regarding the importance and proper use of nets. Instead, radio played a dominant role for the dissemination of information on ITNs as it has been reported in another study [31]. 

Several studies suggest that perceived malaria risk and malaria knowledge are important determinants of bed net ownership and use [25, 32, 33]. For instance, knowledge of malaria amongst mothers of under-five children was associated with ITN use for their children [34]. Factors significantly associated with ITN use were women's knowledge of ITNs and women's lack of problem in using ITNs [35]. However, other studies have reported that greater malaria knowledge, education, and wealth are not consistent determinants of net use [36–38]. It is found that while net replacement remains important, the more education about use and care of nets are likely to increase the use of nets by households [39]. It is also worth mentioning that even when knowledge is a predictor of ITN use; it may not assure protection from malaria unless there is proper use and strong adherence.

The limitation of this study was that we used self-reported ownership and use of ITNs by households without verification and net factors such as color, shape, age. One of the strengths of this study is that we investigated an important group of malaria victims. 

## 5. Conclusion

We conclude that there is low ITN ownership and utilization among the households. The majority of them were obtained from governmental health facilities. Enhanced health education and community mobilization efforts should be employed to increase the possession and proper utilization of insecticide-treated bed nets. The government should re-distribute ITNs in the area to increase the coverage and conduct mass education to increase their utilization among the households.

## Figures and Tables

**Table 1:** Sociodemographic characteristics of respondents in Gursum district, Ethiopia, 2008.

Variables	Frequency	Percentage
Sex		
Male	88	26.3
Female	247	73.7
Religion		
Christian	51	15.2
Muslim	284	84.8
Marital status		
Single	16	4.8
Married	287	85.7
Divorced	12	3.6
Widowed	16	4.8
Separated	4	1.2
Occupational status		
Farmer	104	31.0
Student	26	7.8
House wife	149	44.5
Government employee	12	3.6
Merchant	39	11.6
Others*	5	1.5
Educational level		
Illiterate	228	68.1
Read and write only	16	4.8
Primary education (1–6)	40	11.9
Secondary education (7–12)	32	9.6
Above grade 12	19	5.7
Estimated monthly income of the household (USD per month)^$^		
<10.4	133	39.7
10.4–31.14	103	30.7
31.25–51.98	35	10.4
52.08–83.33	38	11.3
>83.33	26	7.8

* Others: sales and services, clerk.

^$^Exchange rate at the time of the study, 1 USD = 9.6 Ethiopian Birr (ETB).

**Table 2:** Mosquito net ownership and utilization among respondents in Gursum district, Ethiopia, 2008.

Variables	Frequency	Percentage
Is there a bed net in your house		
Yes	209	62.4
No	126	37.6
The number of nets in the household		
1 net	87	41.6
2 nets	116	55.5
3 nets and more	6	2.9
Where did you get it?		
Health office	96	45.9
Health center	32	15.3
Health post/clinic	12	5.7
Shops	17	8.1
Other	52	24.9
When did you obtain it (in months)?		
<6 months	16	7.7
6–12 months	60	28.7
13–24 months	90	43.1
>24 months	43	20.6
ITN utilization		
Yes	45	21.5
No	164	78.5

**Table 3:** Malaria knowledge and history in the past one year among respondents in Gursum district, Ethiopia, 2008.

Variables	Frequency	Percentage
Has anyone in your family caught malaria in the past one year?		
Yes	81	24.2
No	254	75.8
If yes, what were the signs and symptoms he/she was experiencing?		
Fever	80	23.9
Chills	77	23.0
Headache	75	22.4
Backache and generalized aching	58	17.3
Convulsion	29	8.7
Loss of consciousness (coma)	26	7.8
Place of treatment among households who faced malaria		
Government health facility	65	81.2
Private clinic	12	15.0
Traditional healer	2	2.5
Buy drug from any shop	1	1.2
Did anyone in your family travel away from home in the last one month?		
Yes	58	17.3
No	277	82.7
Did she/he use ITN while on travel		
Yes	12	3.6
No	323	96.4
Source of information for ITN		
Mass media	148	48.5
Health workers	87	28.5
Kebele leader	16	5.2
Health extensions workers	24	7.97
Neighborhood	30	9.83

**Table 4:** Final logistic regression model for household's ownership of ITNs (with estimated odds ratios) in Gursum district, Ethiopia, 2008.

Variables	Ownership of ITN		Crude OR (95% CI)	*P* value	Adjusted OR (95% CI)	*P* value
	Yes *n* (%)	No *n* (%)				
Occupation						
Farmer	80 (8.3)	24 (19.0)	4.71 (2.18–10.17)		5.11 (1.88–13.94)	
Student	19 (9.1)	9 (7.1)	2.98 (1.09–8.16)	0.001	2.53 (0.71–8.96)	0.026
Housewife	85 (40.7)	64 (50.8)	1.87 (0.93–3.78)		2.10 (0.87–5.06)	
Government employee	8 (3.8)	5 (4.0)	2.26 (0.63–8.11)		1.46 (0.27–7.91)	
Merchant	17 (8.1)	24 (19.0)	1.00		1.00	
Knowledge of the cause of malaria						
Yes	179 (85.6)	77 (61.1)	3.80 (2.24–6.43)	0.000	2.64 (1.31–5.30)	0.007
No	30 (14.4)	49 (38.9)	1.00		1.00	
Feeling family is at risk of getting malaria						
Yes	111 (55.8)	84 (70.6)	0.53 (0.32–0.85)	0.009	0.33 (0.17–0.69)	0.001
No	88 (44.2)	35 (29.4)	1.00		1.00	
Have you ever heard about ITN						
Yes	199 (97.5)	106 (88.3)	5.26 (1.84–15.0)	0.002	7.56 (1.95–29.28)	0.003
No	5 (2.5)	14 (11.7)	1.00		1.00	
Family size						
Less than 3	38 (18.2)	56 (44.4)	0.20 (0.11–0.38)		0.23 (0.10–0.52)	
Four	36 (17.2)	22 (17.5)	0.49 (0.24–0.99)	0.000	1.05 (0.41–2.68)	0.001
Five and six	58 (27.8)	25 (19.8)	0.69 (0.36–1.34)		0.81 (0.36–1.82)	
Seven and above	77 (36.8)	23 (18.3)	1.00		1.00	

**Table 5:** Final logistic regression model for household's ITNs use (with estimated odds ratios) in Gursum district, Ethiopia, 2008.

Variables	ITN Utilization status		Crude OR (95% CI)	*P* value	Adjusted OR (95% CI)	*P* value
	Yes *n* (%)	No *n* (%)				
Ever heard about ITN in the last 6 months						
Yes	28 (63.6)	49 (31.0)	3.89 (1.93–7.84)	0.000	3.25 (1.50–7.06)	0.003
No	16 (36.4)	109 (69.0)	1.00		1.00	
Family size						
≤4	25 (55.6)	49 (29.9)	2.93 (1.49–5.77)	0.002	2.32 (1.06–5.05)	0.034
>4	20 (44.4)	115 (70.1)	1.00		1.00	
Occupation						
Farmer	7 (15.6)	73 (44.5)	0.11 (0.03–0.37)		0.14 (0.04–0.50)	
Student	9 (20.0)	10 (6.1)	1.01 (0.27–3.75)		0.75 (0.18–3.08)	
Housewife	16 (35.6)	69 (42.1)	0.26 (0.09–0.78)	0.000	0.26 (0.08–0.82)	0.006
Government employee	5 (11.1)	3 (1.8)	1.87 (0.34–10.46)		0.93 (0.15–5.82)	
Merchant	8 (17.8)	9 (5.5)	1.00		1.00	
